# Supplementary material for: Sequencing the orthologs of human autosomal forensic short tandem repeats provides individual- and species-level identification in African great apes
Source: BMC Ecol Evol. 2024 Oct 31;24:134. doi: 10.1186/s12862-024-02324-0 (PMC11526555; doi:10.1186/s12862-024-02324-0)
Supplement: Supplementary file 2 — Supplementary Material 2. [file 12862_2024_2324_MOESM2_ESM.pdf]

```
                                                                    Set 3R-rc
Human        GATCCTCAAAATATGATTGATTTTAAGCCTTATGAGATAATTGTGAGGTCTTAAAATCTG 60
Gorilla      GATCCTCAAAATATGATTGATTTTAAGCCTTATGAGATAATTGTGAGGTCTTAAAATCTG 60
Chimp        GATCCTCAAAATATGATTGATTTTAAGCCTTATGAGATAATTGTGAGGTCTTAAAATCTG 60
Bonobo       GATCCTCAAAATATGATTGATTTTAAGCCTTATGAGATAATTGTGAGGTCTTAAAATCTG 60
             ************************************************************

                                                               Set 1R-rc
Human        AGGTATCAAAACTCAGAGGGAATATATATTCTTAAGAATTATAACGATTCCACATTTAT 120
Gorilla      AGGTATCAAAACTCAGAGTGAATATATATTCTTAAGAATTATAATGATTCCACATTTAT 120
Chimp        AGGTATCAAAACTCAGAGTGAATATATATTCTTAAGAATTATAACGATTCCACATTTAT 120
Bonobo       AGGTATCAAAACTCAGAGTGAATATATATTCTTAAGAATTATAACGATTCCACATTTAT 120
             ****************** ************************* ***************

Human        CCTCATTGACAGAATTGCACCAAATATTGGTAATTAAATGTTTACTATAGACTATTTAGT 180
Gorilla      CCTCATTGACAGAATTGCACCAAATATTGGTAATTAAATGTTTACTATGGACTATTTAGT 180
Chimp        CCTCATTGACAGAATTGCACCAAATATTGGTAATTAAATGTTTACTATAGACTATTTAGT 180
Bonobo       CCTCATTGACAGAATTGCACCAAATATTGGTAATTAAATGTTTACTATAGACTATTTAGT 180
             ************************************************ ***********

Human        GAGATTAAAAAAAACTATCAATCTGTCTATCTATCTATCTATCTATCTATCTATCTATCT 240
Gorilla      GAGATCAAAAAAAAAACTATCTATCCATCTATCTATCTATCTATCTATATCTATCTATCT 240
Chimp        GAGATCAAA-AAA-C--------TATCAATCTGTCTATCTATCTATCTATCTATCTATCT 230
Bonobo       GAGATCAAAAAAA-C--------TATCAATCTGTCTATCTATCTATCTATCTATCTATCT 231
             ***** *** ***          *     * * * * * * * * * *************

                                 Set 3F-rc
Human        AT----CTATCTATCTATCT-ATCGTTAGTTCGTTCTAAACTATGACAAGTGTTCTATCA 295
Gorilla      ATCTATCTATCTATCTATCTATCTGTTAGTTCGTTCTAAACTATGCCAAGTGTTCTATTA 300
Chimp        AT----CTATCTATCTATCT-ATCGTTAGTTCGTTCTAAACTATGCCAAGTGTTCTATCA 285
Bonobo       AT----CTATCTATCTATCT-ATCGTTAGTTCGTTCTAAACTATGCCAAGTGTTCTATCA 286
             **    **************    ********************* ************ *

                                                 Set 1F-rc
Human        TACCCTTTATATATATTAACCTTAAAATAACTCCATAGTCAGCCTGACCAACATGGTGAA 355
Gorilla      TACCCTTTATATATATTAACCTTAAAATAACTCCATAGTCAGCCTGACCAACATGGTGAA 360
Chimp        TACCCTTTATATATATTAACCTTAAAATAACTCCATAGTCAGCCTGACCAACATGGTGAA 345
Bonobo       TACCCTTTATATATATTAACCTTAAAATAACTCCATAGTCAGCCTGACCAACATGGTGAA 346
             ************************************************************

Human        ACCCCGTCTCTAAAAAAAAATACAAAAATTAGCTGGATGCAGTAGCACATGCCTGTAGTCC 415
Gorilla      ACCCCGTCTCTAAAAAAAAATACAAAAATTAGCTGGATGCAGTGGCACATGCCTGTAGTCC 420
Chimp        ACCCCGTCTCTAAAAAAAAATACAAAAATTAGCTGGATGCAGTGGCACATGCCTGTAGTCC 405
Bonobo       ACCCCGTCTCTAAAAAAAAATACAAAAATTAGCTGGATGCAGTGGCACATGCCTGTAGTCC 406
             ****************************************** *****************

Human        CAGCTACTCAGGAGGCTGGGGCAGGAGAACCACTTGACCCAAGA   459
Gorilla      CGGCTACTCAGGAGGCTGGGGCAGGAGAACCACTTGAACCCAA-   463
Chimp        CAGCTACTCAGGAGGCTGGGGCAGGAGAACCACTTGACCCAAGA   449
Bonobo       CAGCTACTCAGGAGGCTGGGGCAGGAGAACCACTTGACCCAAGA   450
             * *********************************** ** *
```

Figure S1: 4-way sequence alignment around D7S820.

Two sets of primers are highlighted (from: https://strbase-archive.nist.gov/str_D7S820.htm)

Set 1F: ATGTTGGTCAGGCTGACTATG; Set 1R: GATTCCACATTTATCCTCATTGAC

Set 3F: TGTCATAGTTTAGAACGAACTAACG; Set 3R: CTGAGGTATCAAAAACTCAGAGG

Primer 3R terminates on a human-specific nucleotide (red font), which may explain the failure to amplify in non-human great apes if the ForenSeq kit employs this primer.

Sequences used are as follows:

Human: hg38 chr7:84160017-84160477; Chimp: panTro5 chr7:86992258-86992710; Bonobo: panPan2 chr7:89792338-89792790; Gorilla: gorGor5 CYUI01015169v1:7435606-7436068

rc: reverse complement

```
Human           GGCAGAATTGCTGGAACCCGGGAAGCTGAGGTCGCAGTGAGCCGAGTTCGCCCCATTGCA 60
Gorilla         GGCAGAATTGCTGGAACCCGGGAAGCCGAGGTTGCAGTGAGCCGAGATCGCCCCGTTGCA 60
Chimp           GGCAGAATTGCTGGAACCCGGGAAGCCGAGGTTGCAGTGAGCCGAGATCGCCCCATTGCA 60
Bonobo          GGCAGAATTGCTGGAACCCGGGAAGCCGAGGTTGCAGTGAGCCGAGATCGCCCCATTGCA 60
                ************************* ***** ************* ******* *****

Human           CTTCAGCCTGGGCTTCGCACCAAGACTCCATTTAAAAAAAA--AATTTTGTTTTAAATAG 118
Gorilla         CTTCAGCCTGGGCTTCGCACCAAGACTCCATTTAAAAAAAATTTTTTTTGTTTTAAATAG 120
Chimp           CTTCAGCCTGGGCTTCGCACCAAGACTCCATTTAAAAAAAAA-AATTTTGTTTTAAATAG 119
Bonobo          CTTCAGCCTGGGCTTCGCACCAAGACTCCATTTAAAAAAA---AAATTTGTTTTAAATAG 117
                ****************************************      **************

Human           TAATGCATTTGTGATAGGTACAGTAGCAATATAAAAAGGAAGATCTGATATTCACATATC 178
Gorilla         TAATGCATTTGTGATAGGTACAGTAGCAATATAAAAAGGAAGATCTGATATTCACATATT 180
Chimp           TAATGCATTTGTGATAGGTACAGTAGCAATATAAAAAGGAAGATCTGATATTCACATATC 179
Bonobo          TAATGCATTTGTGATAGGTACAGTAGCAATATAAAAAGGAAGATCTGATATTCACATATC 177
                ***********************************************************

                                  Forward primer
Human           TCATTGTATTGCTTATTTGTGGGGGTATTTCA-------AGATAACTGTAGATAGGTAG- 230
Gorilla         TCATTGTATTGCTTATTTGTGGGGGAGTCTCGTATTTCAAGATAACTGTAGATAGATAGA 240
Chimp           TCATTGTATTGCTTATTTGTGGGGGAGTCTCGTATTTCAAGATAACTGTAGATAGATAGA 239
Bonobo          TCATTGTATTGCTTATTTGTGGGGGAGTCTCGTATTTCAAGATAACTGTAGATAGATAGA 237
                *************************  * **        **************** ***

Human           --------------------ATAGATAGATAGATAGATAGATAGATAGATAGATAGATAGA 271
Gorilla         TAGATAGATAGA----------------------------TAGATAGATAGATAGATAGA 272
Chimp           TAGACAGATAGATAGATAGATAGACAGATAGATAGATAGATAGATAGATAGATAGATAGA 299
Bonobo          TAGATAGATAGACAGATAGATAGATAGATAGATAGATAGATAGATAGATAGATAGATAGA 297
                                                        ********************

                          Reverse primer
Human           TAGATATTAATAGGTAAACTAGAAGCTTTCAGAAGCTAAATTGGTGTCTTTGGGTGCTCG 331
Gorilla         TAGATATTAATAGGTAAACTAGAAGCTTCCAGAAGCTAAATTGGTGTCTTTGGGTGCTCA 332
Chimp           TAGATATTAATAGGTAAACTAGAAGCTTTCAGAAGCTAAATTGGTGTCTTTGGGTGCTCG 359
Bonobo          TAGATATTAATAGGTAAACTAGAAGCTTTCAGAAGCTAAATTGGTGTCTTTGGGTGCTCG 357
                **************************** ******************************

Human           GTGCGTAATCAGTGAAATCATAATGCCATATCACCTGCCAGGAATACTAAAGTATTTGAA 391
Gorilla         GTGCGTAGTCAGTGAAATCATAATGCCATATCACCTGCCAGGAATACTAAAGTATTTGAA 392
Chimp           GTGCGTAATCAGTGAAATCATAATGCCATATCACCTGCCAGGAATACTAAAGTATTTGAA 419
Bonobo          GTGCGTAATCAGTGAAATCATAATGCCATATCACCTGCCAGGAATACTAAAGTATTTGAA 417
                ******* ****************************************************

Human           ATAAGCTCTCAGACAAGAAATAATAAATGCCGTTAAAAATAGTTTTTATTTCCAACAACC 451
Gorilla         GTAAGCTCTCAGGCAAGAAATAATAAATGCCATTAAAAATAGTTTTTATTTCCAGCAACC 452
Chimp           GTAAGCTCTCAGGCAAGAAATAATAAATGCCATTAAAAATAGTTTTTATTTCCAACAACC 479
Bonobo          GTAAGCTCTCAGGCAAGAAATAATAAATGCCATTAAAAATAGTTTTTATTTCCAACAACC 477
                 *********** ****************** ********************** *****

Human           ATTCAGATAATTTAGCAACCGTT    474
Gorilla         ATTCAGATAATTTAGCAACCGTT    475
Chimp           ATTCAGATAATTTAGCAACCGTT    502
Bonobo          ATTCAGATAATTTAGCAACCGTT    500
                ***********************
```

Figure S2: 4-way sequence alignment around D9S1122.

The primers highlighted are from Hill et al. (2009) J. Forens. Sci. 54, 1008-1015; doi: 10.1111/j.1556-4029.2009.01110.x with sequences F: GGGTATTTCAAGATAACTGTAGATAGG; R: GCTTCTGAAAGCTTCTAGTTTACC.

The forward primer lies over a human-specific 7-bp indel (red font), which may explain the failure to amplify in non-human great apes if the ForenSeq kit employs this primer.

Sequences used are as follows:

Human: hg38 chr9:77073597-77074072**;** Chimp: panTro5 chr9:54320898-54321400**;** Bonobo: panPan2 chr9:75928729-75929231**;** Gorilla: gorGor5 CYUI01015053v1:6721004-6721478

```
Human        AGTCTTCTTTTTCTAAACCTCTCCCATCTGGATAGTGGACCTCATATTTCAGATGCTAAT   60
Gorilla      AGTCTTCTTTTTCTAAACCTCTCCCATCTGGATAGTGGACCTCATATTTCAGATGCTAAT   60
Chimp        AGTCTTCTTTTTCTAAACCTGTCCCATCTGGATAGTGGACCTCATATTTCAGATGCTAAT   60
Bonobo       AGTCTTCTTTTTCTAAACCTGTCCCATCTGGATAGTGGACCTCATATTTCAGATGCTAAT   60
             ******************** ***************************************

Human        AGGCTGTTGAGGTAGTTTCCTAAGCAAAAAAGTAATTGTCTCTCTCAGAGGAATGCTTTA   120
Gorilla      AGGCTGTTAAGGTAGTTTCCTAAGCAAAAAAGTAATTGTCTCTCTCAGAGGAATGCTTTA   120
Chimp        AGACTGTTGAGGTAGTTTCCTAAGCAAAAAAGTAATTGTCTCTCTCAGAGGAATGCTTTA   120
Bonobo       AGGCTGTTAAGGTAGTTTCCTAAGCAGAAAAGTAATTGTCTCTCTCAGAGGAATGCTTTA   120
             **.*****.*****************.*********************************

                            Set 2R-rc        Set 1R-rc
Human        GTGCTTTTTAGCCAAGTGATTCCAATCATAGCCACAGTTTACAACATTTGTATCTTTATC   180
Gorilla      GTGCTTTTTAGCCAAGTGATTCCAATCATAGCCACAGTTTACAACATTTGTATCTTTATC   180
Chimp        GTGCTTTTTAGCCAAGTGATTCCAATCATAGCCACAGTTTACAACATTTGTATCTTTATC   180
Bonobo       GTGCTTTTTAGCCAAGTGATTCCAATCATAGCCACAGTTTACAACATTTGTATCTTTATC   180
             ************************************************************

Human        TGTATCCTTATTTATACCTCTATCTATCTATCTATCTATCTATCTATCTATCTATCTATC   240
Gorilla      TGTATCTTTATTTATACCTCTCTCTCTC----------------------TCTCTCTATC   220
Chimp        TGTATCCTTATTTATACCTCTCTC----------------------TCTATCTATCTATC   218
Bonobo       TGTATCCTTATTTATACCTCTCTC----------------------TCTGTCTATCTATC   218
             ****** **************.**                          ***.******

           Pan-specific variant   Set 1F-rc  Set 2F-rc
Human        TATCTTCAAAATATTACATAAGGATACCAAAGAGGAAAATCACCCTTGTCACATACTTGC   300
Gorilla      TATCTTCAAAATATTACATAAGGATACCAAAGAGGAAAATCACCCTTGTCACATACTTGC   280
Chimp        TATCTTCAAAATATTACGTAAGGATACCAAAGAGGAAAATCACCCTTGTCACATACTTGC   278
Bonobo       TATCTTCAAAATATTACGTAAGGATACCAAAGAGGAAAATCACCCTTGTCACATACTTGC   278
             *****************.******************************************

Human        TATTAAAATATACTTTTATTAGTACAGATTATCTGGGACACCACTTTAATTAGAAGCTTT   360
Gorilla      TATTAAAATATACTTTTGTTAGTACAGATTATCTGGGACACCACTTTAATTAGAAGCTTT   340
Chimp        TATTAAAATATACTTTTATTAGTACAGATTATCTGGGACACCACTTTAATTAGAAGCTTT   338
Bonobo       TATTAAAATATACTTTTATTAGTACAGATTATCTGGGACACCACTTTAATTAGAAGCTTT   338
             *****************.******************************************

Human        AAAAGCATATGCATGTCTCAGTATTTAATTTAAAATTATTACATAATTATATACTCCTT   420
Gorilla      AAAAGCATATGCATGTCTCAGTATTTAATTTAAAATTATTACATAATTATGTACTCCTT   400
Chimp        AAAAGCATATGCGTGTCTCAGTATTTAATTTAAAATTATTACATAATTATGCACTCCTT   398
Bonobo       AAAAACATATGCATGTCTCAGTATTTAATTTAAAATTATTACATAATTATGCACTCCTT   398
             ****.*******.**************************************. *******

Human        TGAATTAGAA   430
Gorilla      TGAATTAGAA   410
Chimp        TGAATTAGAA   408
Bonobo       TGAATTAGAA   408
             **********
```

Figure S3: 4-way sequence alignment around D5S818.

Two sets of primers are highlighted (from: https://strbase-archive.nist.gov/str_D5S818.htm)

Set 1F: GGTGATTTTCCTCTTTGGTATCC; Set 1R: AGCCACAGTTTACAACATTTGTATCT

Set 2F: GGGTGATTTTCCTCTTTGGT; Set 2R: TGATTCCAATCATAGCCACA

None of these primers is predicted to be affected by Pan-specific variants. However, primers 1F and 2F terminate close to a Pan-specific variant (red font) that, if included in the ForenSeq primer, would explain the failure to amplify in Pan.

Sequences used are as follows:

Human: hg38 chr5:123775355-123775784; Chimp: panTro5 chr5:123606425-123606832; Bonobo: panPan2 chr5:124943119-124943526; Gorilla: gorGor5 CYUI01015135v1:125136-125545

rc: reverse complement

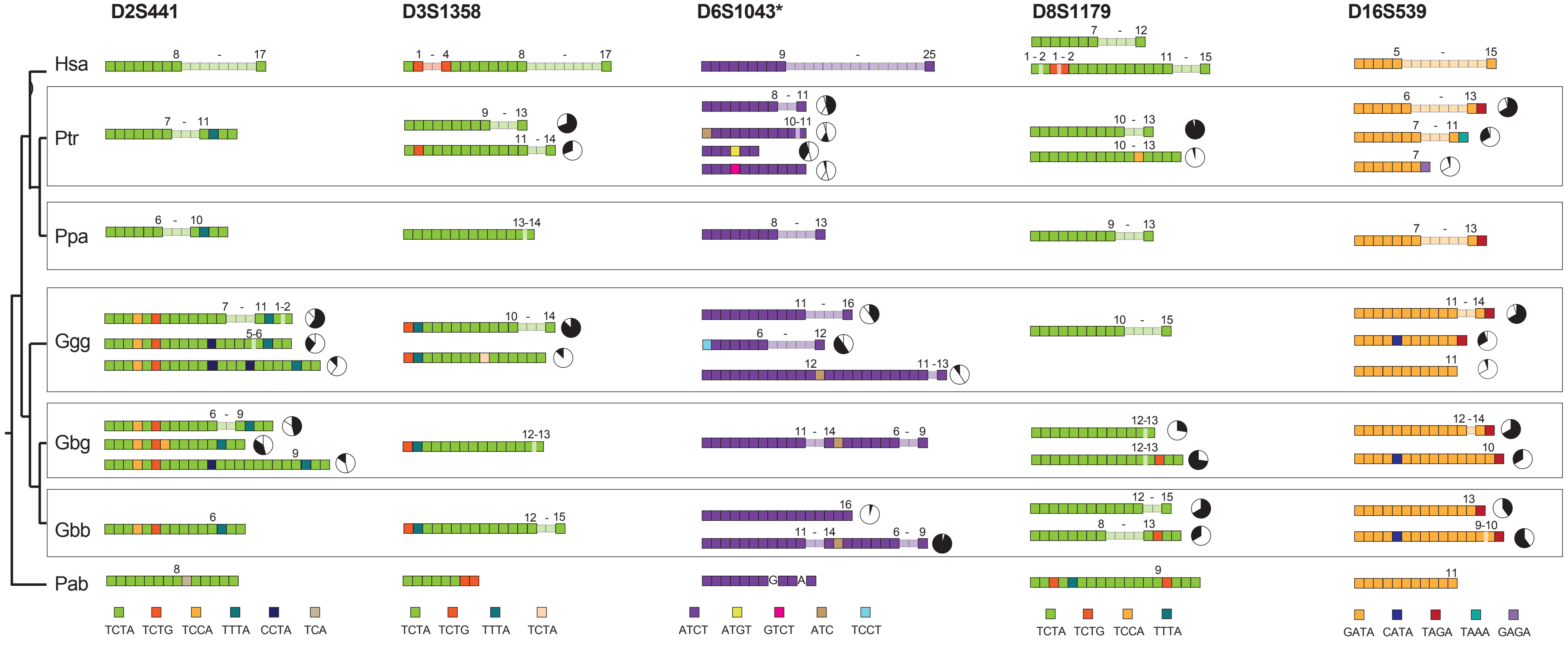

Figure S4: Summary of inter- and intra-specific structural variation at 10 STRs (*continued on next page*).

Schematic representation of variation across (sub)species, phylogenetically arranged, for ten STRs (see also Fig. 4). Human structures are from strbase.nist.gov and Gettings et al. (2016); for FGA, two highly divergent structures are shown. Orthologous orangutan (Pab: *Pongo abelii*) alleles are based on the reference sequence; none is observed for FGA. In each case, tetra- or tri-nucleotide repeat motifs are indicated by coloured boxes. Ranges of repeat numbers within variable arrays are indicated. Where more than one structural class is observed within *Pan* or *Gorilla*, pie-charts indicate their proportions. Hsa: *Homo sapiens*; Ptr: *Pan troglodytes*; Ppa: *P. paniscus*; Ggg: *Gorilla gorilla gorilla*; Gbg: *G. beringei graueri*; Gbb: *G. b. beringei*.

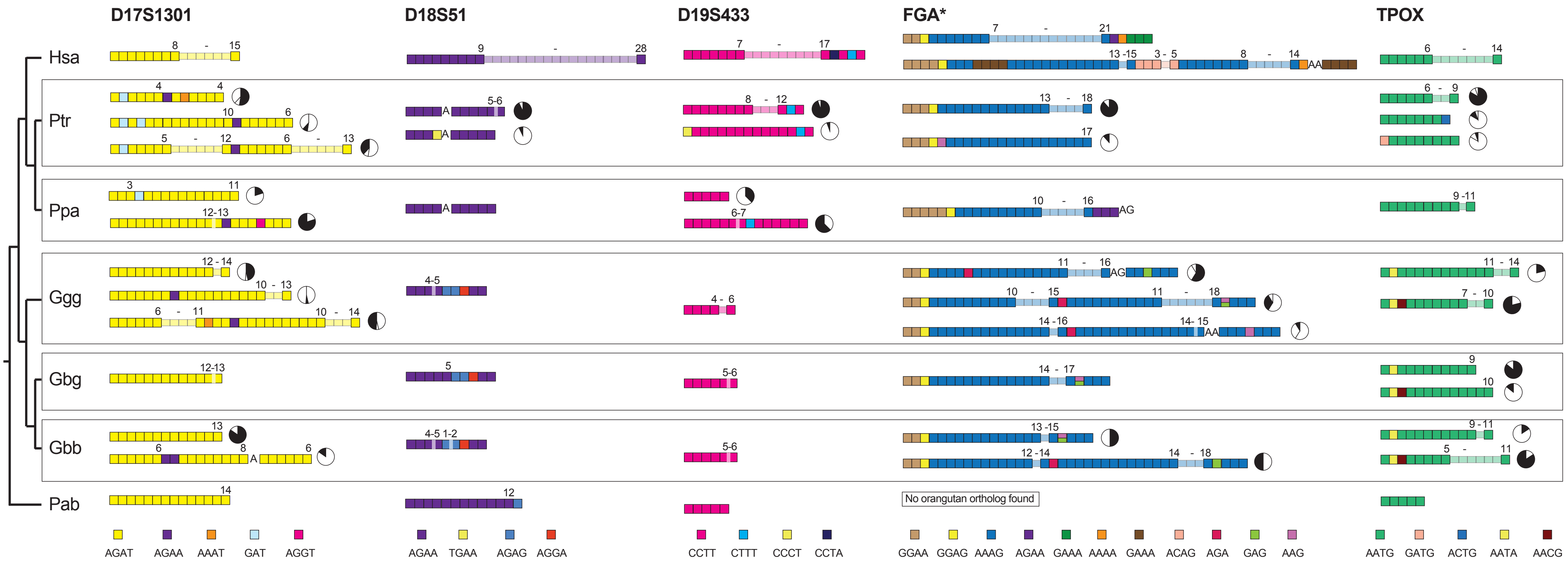

Figure S4: Summary of inter- and intra-specific structural variation at 10 STRs (*continued from previous page*).

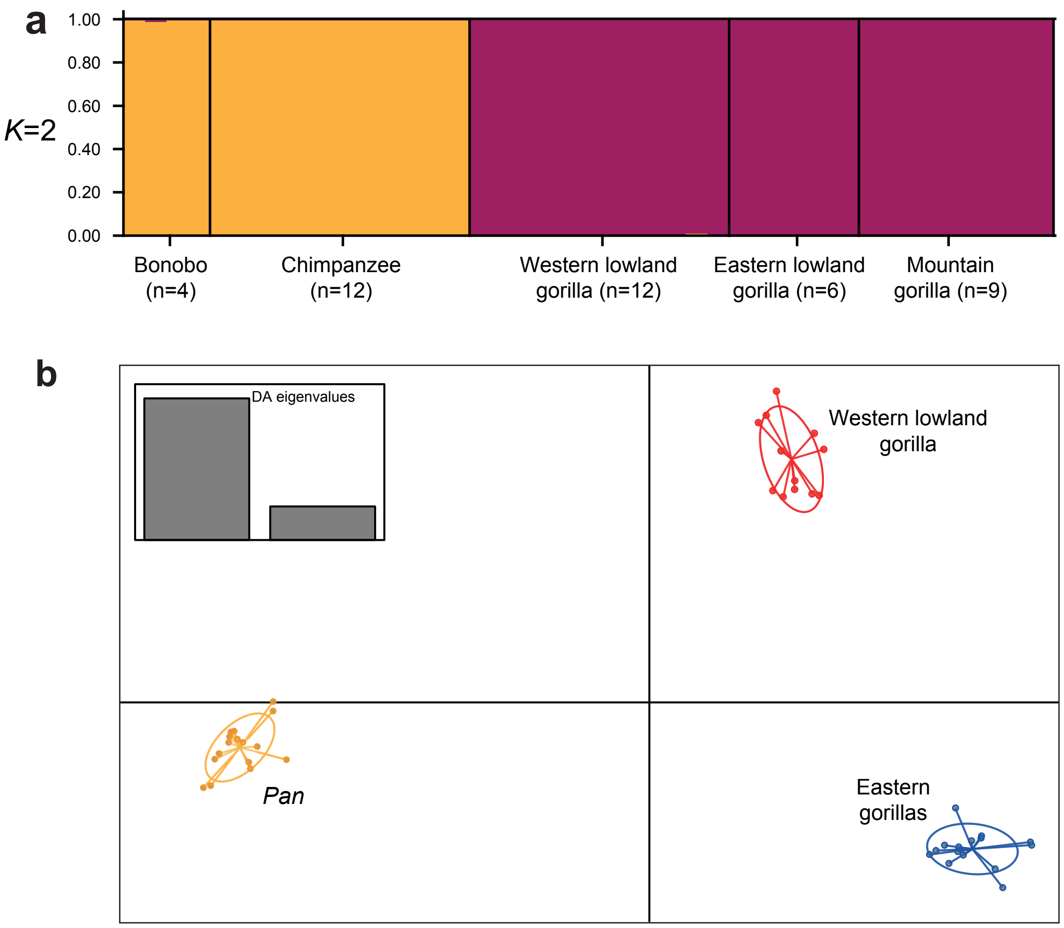

Figure S5: Cluster analysis based on CE-equivalent autosomal STR genotypes.

a) Results based on STRUCTURE, for *K*=2; b) Results based on DAPC analysis. Full information from MPS data was used here. An analysis based on sequence data (both array and flanking sequence variation) is shown in Figure 5. Related individuals are removed for this analysis (see Table S1).
